# Effect of Silver Nanoparticles on pH-Indicative Color Response and Moisture Content in Intelligent Films Based on Peruvian Purple Potato and Polyvinyl Alcohol

**DOI:** 10.3390/polym17111490

**Published:** 2025-05-27

**Authors:** Antony Alexander Neciosup-Puican, Carolina Parada-Quinayá

**Affiliations:** 1 Facultad de Ciencias, Universidad Nacional de Ingeniería, Av. Tupac Amaru 210, Lima 15333, Peru; 2 Bioengineering and Chemical Engineering Department, Universidad de Ingeneria y Tecnologia—UTEC, Lima 15063, Peru; dparada@utec.edu.pe; 3 Bioengineering Research Center—BIO, Universidad de Ingenieria y Tecnologia—UTEC, Lima 15063, Peru

**Keywords:** silver nanoparticles, food packaging, purple potato extract, anthocyanin, pH sensors

## Abstract

The growing need for sustainable packaging materials with enhanced functionality has prompted our investigation into biodegradable polymers reinforced with nanostructures. In this work, we began by extracting anthocyanins from pigmented native potatoes (*Solanum tuberosum*) and confirming their concentration via UV–Visible spectroscopy. The corresponding potato starch was then characterized according to its amylose and amylopectin contents. The natural pigments subsequently served as reducing and stabilizing agents in a green synthesis of silver nanoparticles (AgNPs), which were subsequently incorporated into starch matrices derived from the same tuber. To evaluate the performance of the resulting composite films, we examined their pH-responsive color behavior—demonstrating their potential as visual indicators—their molecular structure through FTIR analysis—to verify the successful integration of AgNPs—and their moisture content as a measure of barrier properties. The AgNP-containing films exhibited markedly improved color stability across varying pH levels and superior moisture retention compared to pure starch films. These results illustrate the promise of combining underutilized Andean crops with eco-friendly nanotechnology to produce advanced, biodegradable materials suitable for intelligent food-packaging applications.

## 1. Introduction

The development of functional polymeric materials has gained significant attention due to their potential applications in biomedical, food packaging, and environmental fields [1,2,3]. Among these materials, natural biopolymers such as starch have emerged as sustainable alternatives to synthetic polymers, owing to their biodegradability, biocompatibility, and widespread availability [4,5]. However, the inherent limitations of starch-based films, such as poor mechanical strength and thermal instability, have prompted the design of blended polymeric matrices [6,7].

Recent advancements in intelligent packaging have explored the development of pH-sensitive films utilizing blends of different biodegradable polymers. Ke et al. (2024) investigated the incorporation of anthocyanins from purple potato into chitosan/polyvinyl alcohol/nano-ZnO matrices [8]. The resulting films exhibited enhanced pH responsiveness and antibacterial properties, further broadening the scope of potato-based pH-sensitive films in intelligent packaging solutions [8]. Further research by Wang et al. (2023) focused on enhancing the stability and functionality of such films [9]. They incorporated anthocyanins, extracted from purple sweet potato, into a composite film matrix, resulting in improved pH sensitivity and mechanical properties. The optimized film demonstrated reliable performance in monitoring pork freshness and preserving cherry quality [9].

One promising strategy to enhance the limited functional properties involves the incorporation of bioactive agents, particularly natural anthocyanins, and silver nanoparticles (AgNPs). Choi et al. (2017) developed a colorimetric pH indicator film composed of agar, potato starch, and anthocyanin extracts from purple sweet potato (*Ipomoea batatas*) [10]. The film exhibited distinct color changes across a pH range of 2.0 to 10.0, transitioning from red in acidic conditions to green in alkaline environments. This property enabled the film to function effectively as a meat spoilage sensor, indicating freshness through visible color shifts [10].

Silver nanoparticles are well known for their broad-spectrum antimicrobial activity, high surface area, and tunable physicochemical properties [11]. Silver nanoparticles (AgNPs) have emerged as versatile additives in polymer matrices, not only due to their well-documented antimicrobial activity, but also for their capacity to modify and enhance the optical properties of polymers. This enhancement is primarily attributed to localized surface plasmon resonance (LSPR), which imparts unique optical behaviors, such as dichroism and environmental sensitivity, to AgNPs [12].

Green synthesis of AgNPs using plant-based extracts rich in phenolic compounds offers an eco-friendly approach that avoids the use of toxic reducing agents and harsh reaction conditions [13]. In this context, anthocyanin-rich extracts from pigmented native potatoes (*Solanum tuberosum*) serve not only as natural reducing and stabilizing agents during nanoparticle synthesis but also confer antioxidant properties that may enhance the functional performance of the resulting materials [13,14].

A recent study investigated the incorporation of polymorphic AgNPs into a polyvinyl alcohol (PVA) matrix. The results demonstrated that the nanoparticles retained their dichroic properties upon embedding in the polymer. Furthermore, the morphology of the AgNPs directly influenced the color of the final material: triangular nanoparticles produced a blue hue, spherical particles resulted in yellow, and irregular shapes generated reddish tones [12]. These stable optical characteristics open up new opportunities in optical filters, decorative coatings, and smart materials.

Accordingly, AgNPs have also been successfully incorporated into polymer-based systems for contaminant detection [15]. Abbasi et al. (2022) reported a starch-stabilized AgNPs colorimetric sensor for selective Hg^2+^ detection. The system exhibited a visible color change from yellow to colorless, attributed to a redox reaction between the AgNPs and mercury ions, enabling direct visual detection [16].

Collectively, these studies demonstrate that the integration of silver nanoparticles into polymer matrices not only improves colorimetric properties, but also imparts multifunctionality, including sensing capability, antimicrobial activity, and enhanced thermal stability. These features position AgNP-based polymer composites as promising candidates for active packaging, smart textiles, and environmental monitoring systems.

The key scientific question we address, therefore, is whether anthocyanin-derived, plasmonically active AgNPs can simultaneously enhance color stability and moisture management in starch/PVA films, thereby transforming a fragile biopolymer into a multifunctional, intelligent packaging material.

To answer this question, we synthesize AgNPs using anthocyanin extracts from pigmented native potatoes and embed them into potato-starch/PVA matrices.

The effects of this incorporation on color stability and moisture of the resulting materials are evaluated. This work not only highlights the valorization of underutilized Andean agrobiodiversity, but also contributes to the development of sustainable, functional materials with potential applications in active packaging and biomedical systems.

## 2. Materials and Methods

### 2.1. Materials

Peruvian purple potatoes (INIA 328–*Kulli papa*) were purchased from a local farmers’ market in Jangas, Ancash province, Peru.

Analytical grade silver nitrate (Merck (Rahway, NJ, USA), 99.9%), sodium hydroxide (Merck (Rahway, NJ, USA), ≥98%), ethanol (Baker Analyzed (Phillipsburg, NJ, USA), 99.9%), citric acid (Merck (Rahway, NJ, USA), 99.5%), potassium chloride (Merck (Rahway, NJ, USA), 99.9%) and sodium acetate trihydrate (Merck (Rahway, NJ, USA), 99.9%) were purchased commercially.

Colloidal silver nanoparticles (AgNPs) were synthesized using anthocyanin extract obtained from Peruvian purple potatoes, following the methodology reported in a previous study [13].

### 2.2. Extraction and Quantification of Anthocyanins

Peruvian purple potatoes were thoroughly washed and cut into uniform cubes measuring 2 cm × 2 cm. These pieces were then subjected to freezing at −40 °C before being lyophilized under dark conditions to prevent any degradation of sensitive compounds. The freeze-dried potatoes were subsequently ground into a fine powder and sieved using a 140-mesh sieve, yielding a purple potato powder.

To extract anthocyanins from the purple potato powder, a conventional extraction method utilizing ethanol 40% *v*/*v* was employed. This method was based on optimal conditions identified in a previous study [17]. Specifically, the extraction process was carried out at a controlled temperature of 40 °C, with a solvent-to-solid ratio of 5 mL of solvent per gram of purple potato powder. The extraction period lasted for 4 h, ensuring maximum yield of anthocyanins. The total anthocyanin content was determined using the differential pH method, which relies on the structural changes in anthocyanin chemical forms and their absorbance measurements at pH 1.0 and 4.5. For this analysis, 0.1 mL of the extract was diluted separately with 7.9 mL of 0.025 mol/L potassium chloride buffer (pH 1) and 9 mL of 0.4 mol/L sodium acetate buffer (pH 4.5). The total anthocyanin content (TA) was quantified and expressed as cyanidin-3-glucoside equivalents, using the following Equation (1):

(1) $$TA\left(\frac{mg}{L}\right)=\frac{A\times MW\times FD\times 1000}{\epsilon \times l}$$

where $A={\left(A_{vis-max}-A_{\lambda \;700}\right)}_{pH\;1}-{\left(A_{vis-max}-A_{\lambda \;700}\right)}_{pH\;4.5}$; MW is the molecular weight of cyanidin 3-O-glucoside (449.2 g/mol); FD is the extract dilution factor; ϵ is the molar extinction coefficient (26,900 L/mol·cm); l is the path length in cm; and 1000 is the conversion factor from gram to milligram.

### 2.3. Color Evaluation of Anthocyanin Extract

According to Reyes and Cisneros-Zevallos (2007) [18], 8 mL of buffer solution were prepared with pH values ranging from 1 to 10. Subsequently, 200 µL of anthocyanin extract were added and mixed by magnetic stirring for 30 min. Finally, the color of the mixture was analyzed using a colorimeter (PCE-XXM30, PCE Instrument, Meschede, Germany).

### 2.4. Amylose and Amylopectin Content

The amounts of amylose and amylopectin in purple potato powder were determined using a modified version of the method developed by M. Lal et al. (2024) [19]. Initially, 20 mg of starch were weighed into a 50 mL screw-cap tube. Then, 0.2 mL of 95% ethanol and 1.8 mL of 1N sodium hydroxide were added to the tube. The mixture was left to rest at room temperature for 24 h. After this period, the volume was brought up to 20 mL with deionized water, shaken vigorously, and allowed to rest for 30 min. In a volumetric flask, 1 mL of the aliquot was mixed with 2 mL of 1N acetic acid and 0.4 mL of an iodine solution (prepared by dissolving 1 g of iodine and 10 g of KI in 500 mL of water). The total volume was then made up to 20 mL with deionized water. The solution was left in the dark for 20 min for color development and transferred to cuvettes for absorbance measurement using a UV–Visible spectrophotometer at 620 nm.

Amylopectin was calculated by the difference in total starch and amylose using the following Equation (2):

(2) $$Amylopectin\left(\%\right)=100\;\%-amylose\;content(\%)$$

### 2.5. Preparation of Films

#### 2.5.1. Preparation of the Purple Potato Film (P.P.)

The purple potato starch-based film was prepared using the solvent casting method. The process began by preparing an aqueous solution in which 46.5 mL of distilled water was acidified with citric acid until the pH reached 2. The acidification helps to enhance the stabilization of anthocyanins present in the purple potato powder. Once the solution was acidified, 3.5 g of purple potato powder and 1.4 g of sorbitol were added. Sorbitol was used as a plasticizer, which helps to improve the flexibility and mechanical properties of the final film. The mixture was then stirred magnetically at 800 rpm for 30 min at room temperature to ensure proper homogenization and dispersion of the components. Following this, the temperature of the solution was gradually raised to 90 °C and maintained for 15 min. This thermal treatment induced the gelatinization of starch, which is essential for film formation. The gelatinized mass, referred to as the gelatinized purple potato (GPP) matrix, became a more viscous and homogeneous gel-like substance. Once the gelatinization process was complete, 25 g of the hot mixture was poured into polyethylene Petri dishes and evenly spread to form a thin film. The films were then dried in a convection oven at 80 °C for 4 h to remove excess moisture and allow proper film formation.

#### 2.5.2. Preparation of the Purple Potato–PVA Film (P.P–PVA)

To prepare the P.P–PVA film, the gelatinized purple potato mixture (GPP) was blended with a 10% *w*/*w* aqueous PVA solution at a ratio of 70:30 (GPP:PVA, *w*/*w*). The mixture was stirred at room temperature for 1 h to ensure proper integration of the components. Once the solution was well mixed, it was cast and dried under the same conditions as the P.P films.

#### 2.5.3. Preparation of the Purple Potato–PVA–AgNP Composite Film (P.P–PVA–AgNP)

For the P.P–PVA–AgNP film, 1 g of colloidal silver nanoparticles (AgNPs) was first mixed into the 10% *w*/*w* PVA solution before combining it with the GPP matrix. The same 70:30 (GPP:PVA, *w*/*w*) ratio was maintained. The resulting mixture was stirred for 1 h at room temperature and then cast and dried using the same procedure as the other films.

### 2.6. pH-Response of Samples Films

To evaluate the response of the films at different pH levels, we adopted the method developed by Y. Qi and Y. Li (2024) [20]. Film samples were cut into 2 cm × 2 cm pieces and immersed in buffer solutions with pH values of 1, 4, 7, and 10 for 30 min. After immersion, the films were photographed, and the color parameters $L^{*}$ (brightness), $a^{*}$ (−green to +red), and $b^{*}$ (−blue to +yellow) were measured using a colorimeter (PCE-XXM30, PCE Instrument, Meschede, Germany) to determine the effect of pH on the films. The total color difference (ΔE) was calculated using the following Equation (3):

(3) $$\Delta E=\sqrt{{\left(L^{*}-L_{0}\right)}^{2}+{\left(a^{*}-a_{0}\right)}^{2}+{\left(b^{*}-b_{0}\right)}^{2}}$$

where $L^{*}$, $a^{*}$, and $b^{*}$ are the values of the samples; $L_{0}$, $a_{0}$, and $b_{0}$ represent the original values of the standard white plate, and the values of $L_{0}$, $a_{0}$, and $b_{0}$ were 92.7, −1.3, and −4, respectively.

### 2.7. Fourier Transform Infrared (FTIR) Spectrum

The Fourier-transform infrared (FTIR) spectra of the sample films and purple potato powder were obtained using an FTIR spectrophotometer (Model IRTracer-100, Shimadzu, Kyoto, Japan) equipped with an attenuated total reflectance (ATR) accessory (Pike Technologies, Madison, WI, USA). Spectral data were collected over the wavenumber range of 400 to 4000 cm^−1^ at a resolution of 2 cm^−1^. Each sample was placed directly onto the diamond crystal of the ATR module, and sufficient pressure was applied to ensure proper contact. The resulting spectra were used to identify the functional groups and molecular interactions present in the films and raw materials.

### 2.8. Moisture Content

The moisture content of the sample films was determined using a precision moisture analyzer (Model MB 120, Ohaus Corporation, Parsippany, NJ, USA). Approximately 5 g of each film sample were placed in the analyzer and subjected to halogen heating at a constant temperature of 105 °C. The samples were heated until a stable weight was achieved, indicating the complete evaporation of moisture. The moisture content was then automatically calculated by the instrument based on the initial and final sample weights.

## 3. Results

### 3.1. UV–Visible Spectroscopy: Anthocyanin Content and AgNP Surface Plasmon Resonance

**Figure 1:**
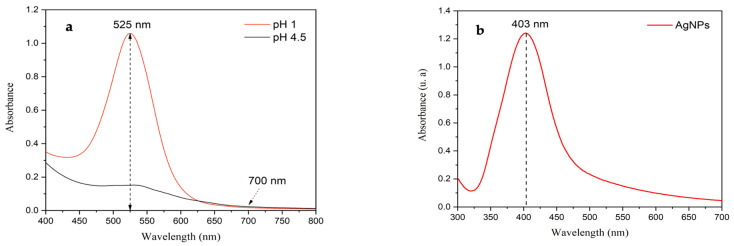
(**a**) UV–Visible spectrum of freeze-dried Peruvian purple potatoes anthocyanin extract at pH 1.0 and pH 4.5 and (**b**) UV–visible spectra of AgNPs.

The anthocyanin content in freeze-dried Peruvian purple potatoes was quantified using the differential pH method, employing UV–Visible spectroscopy in buffer solutions at pH 1 and pH 4.5 (Figure 1a). The analysis revealed an anthocyanin concentration of 153.03 mg/100 g, which is significantly higher than the 75.71 mg/100 g reported in a previous study where fresh potatoes were used under the same extraction conditions [17]. This substantial difference highlights the effectiveness of freeze-drying in preserving anthocyanins, in contrast to the grating process, which causes greater degradation due to enzymatic oxidation and exposure to environmental factors.

Freeze-drying is a more effective method for preserving bioactive compounds such as anthocyanins, as it minimizes degradation by reducing exposure to heat and oxygen, both of which contribute to the breakdown of these sensitive compounds [21]. Additionally, the freeze-drying process removes water at low temperatures, helping to maintain the integrity and concentration of anthocyanins in Peruvian purple potatoes. Consequently, freeze-drying not only preserves these compounds more effectively but also enhances their concentration.

Figure 1b displays the UV–visible spectrum of the biosynthesized silver nanoparticles, featuring the characteristic surface-plasmon-resonance (SPR) absorption maximum at 403 nm. This band corresponds to AgNPs with an average diameter of approximately 21.6 nm, as detailed in our previous work (Neciosup-Puican et al., 2024) [22].

### 3.2. Color Evaluation of Anthocyanin Extract

**Figure 2:**
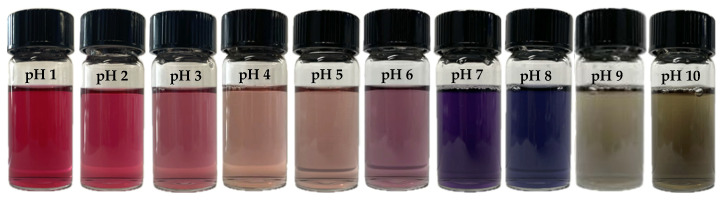
Color of anthocyanin extract in different pH buffer.

Figure 2 shows the color changes of the anthocyanin extract in the pH range of 1 to 10. At pH levels below 4.0, the extract exhibits a red coloration. As the pH increases to 4.0–6.0, the color shifts to pink. At neutral pH 7.0, the extract turns purple, which shifts to blue at pH 8.0, and finally becomes green at pH levels between 9.0 and 10.0. These distinct color changes result from structural modifications in the anthocyanin molecules in response to varying hydrogen ion concentrations. The transformations occur due to shifts in the equilibrium between different anthocyanin forms, such as flavylium cations (red at low pH), quinoidal bases (blue at neutral to slightly alkaline pH), and their associated anionic structures at higher pH levels. This pH-dependent behavior highlights the potential of anthocyanins as natural pH indicators and underscores their sensitivity to environmental conditions [23,24].

Table 1 presents the color parameters (L*, a*, b*, and ΔE) of the anthocyanin extract when subjected to varying pH levels ranging from 1 to 10. The results demonstrate a significant influence of pH on the chromatic properties of the extract, leading to noticeable color shifts due to structural transformations of anthocyanin molecules.

A significant decrease in the value of parameter $a^{*}$ was observed, declining from +41.7 to −1.9, which indicates a shift in the color of the anthocyanin extract from red to green. Additionally, there was a marked increase in the value of parameter $b^{*}$ from −5.2 to +20.6, signifying that the color of the anthocyanin extract transitioned towards yellow.

Changes in brightness were also noted, with lower $L^{*}$ values occurring between pH 6 and pH 8, which appeared visually as the darkest shades. Conversely, higher $L^{*}$ values were observed outside this pH range, indicating greater brightness in those conditions. These variations indicate that the color response of the anthocyanin extract to pH alterations involves complex interactions that affect both hue and lightness. This results in a dynamic and visually discernible color palette.

### 3.3. Amylose and Amylopectin Content

The amylose and amylopectin content are key factors influencing the properties and performance of starch-based thermoplastics. These two polysaccharides differ in their molecular structure and behavior, directly impacting the mechanical, thermal, and barrier properties of starch-based materials.

According to A. Mohammadi Nafchi et al. (2013), a higher amylose content leads to greater rigidity and tensile strength, as amylose molecules have a linear structure that promotes strong intermolecular interactions and film formation [25]. In contrast, higher amylopectin content enhances flexibility and elasticity, due to its highly branched molecular structure, which disrupts ordered packing and increases plasticity [25].

For the purple Peruvian potato, the amylose content was determined to be 23.76%, while the amylopectin content was 76.24%. These values are consistent with those reported in the literature for various potato species [26,27,28]. The moderate amylose content in purple Peruvian potato starch suggests that it can form strong yet somewhat flexible films, making it a promising candidate for applications in biodegradable packaging and starch-based thermoplastics.

Additionally, the high amylopectin content enhances the gelatinization properties, influencing factors such as swelling power, solubility, and retrogradation behavior. This balance between amylose and amylopectin makes purple Peruvian potato starch particularly suitable for film formation, bioplastic applications, and edible coatings where both strength and flexibility are desirable.

### 3.4. pH-Response of Samples Films

Table 2 provides a detailed illustration of the color transformations observed in film samples (P.P, P.P–PVA, and P.P–PVA–AgNP) when immersed in buffer solutions at different pH values (1, 4, 7, and 10), along with their specific color parameters (L*, a*, b*) and total color difference (ΔE).

Initially, the films exhibited predominantly red colors in highly acidic conditions (pH 1). Gradually increasing the pH towards more neutral conditions (pH 4 and 7), the films transitioned through pink and brown shades, ultimately developing a green coloration when immersed in a basic solution (pH 10).

Quantitative analysis indicates that the a* parameter consistently decreases with increasing pH, visually confirming the transition from red to green. This phenomenon is particularly pronounced in the film containing silver nanoparticles (PP–PVA–AgNP), which experienced a significant decrease from 36.1 to −0.1, highlighting remarkable sensitivity and clear signaling of pH changes.

On the other hand, the b* parameter, associated with yellow tones, also shows a considerable increase, particularly in the original P.P film, rising from 3.7 to 17.4, indicating a shift towards more yellowish colors with increasing basicity. However, incorporating PVA introduces an inflection in this trend at higher pH values, an effect positively moderated by the addition of silver nanoparticles.

In terms of luminosity (L*), the original P.P films exhibit slightly lower values, indicating overall lower brightness. However, incorporating PVA significantly enhances brightness, particularly in acidic conditions (pH 1–4), reaching values close to 40.1 at pH 7. Subsequent incorporation of AgNPs maintains this brightness improvement without any negative impacts, providing additional benefits that preserve the visual clarity of the films.

These color changes arise from the pH-dependent equilibria of anthocyanins: red flavylium cations dominate in acid, while blue–green quinoidal bases and yellow chalcones prevail in neutral to basic media. When anthocyanins cap silver nanoparticles, plasmon–chromophore coupling amplifies and stabilizes the corresponding spectral shifts, giving the P.P–PVA–AgNP films a particularly sharp and reversible pH response.

Therefore, incorporating silver nanoparticles considerably enhances the visual characteristics of the films, enabling clear and perceptible signaling of pH changes. This positions P.P–PVA–AgNP films as promising food quality indicators, effectively reflecting storage conditions and food deterioration through distinctive and easily recognizable chromatic changes.

### 3.5. Fourier Transform Infrared (FTIR) Spectrum

**Figure 3:**
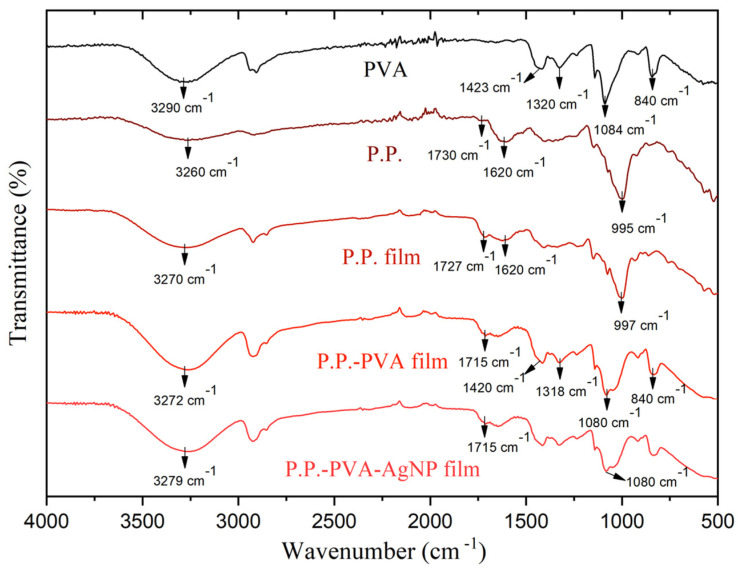
FTIR spectra for pure PVA, pure P.P, P.P film, P.P–PVA film, and P.P–PVA–AgNP films.

The chemical structure of the films was analyzed using Fourier Transform Infrared (FTIR) spectroscopy. Figure 3 presents the FTIR results. The FTIR spectrum of the P.P film reveals a significant absorption band at approximately 3270 cm^−1^, attributed to the stretching vibrations of OH groups, indicative of the presence of hydroxyl functionalities. Furthermore, characteristic absorption bands at 1727 cm^−1^ and 1620 cm^−1^ are clearly observed and correspond to the carbonyl (C=O) and carbon–carbon double bond (C=C) vibrations, respectively. These bands highlight the presence of anthocyanins within the P.P film [29]. Additional notable absorption features are present at 1210 cm^−1^, associated with C-O bond vibrations characteristic of anthocyanins, and at 995 cm^−1^, linked to C-O-C ring vibrations from starch content present in the pure P.P material [30].

Upon blending P.P with PVA to form the P.P–PVA film, spectral contributions from both individual components become clearly evident. This composite material exhibits characteristic PVA absorption bands at 1420 cm^−1^, associated with CH bending vibrations, 1318 cm^−1^ corresponding to CH deformation, 1080 cm^−1^ related to CO stretching vibrations, and 840 cm^−1^ linked to C-C group vibrations [31,32]. These bands confirm the successful integration of both polymeric materials into a homogenous blend.

It is noteworthy that the FTIR spectrum of the P.P–PVA–AgNP film closely resembles that of the P.P–PVA film. This similarity is attributed to the inert nature of silver nanoparticles (AgNPs), which, being metallic, do not possess functional groups capable of producing distinct bands in FTIR spectroscopy [33].

### 3.6. Moisture Content

The moisture content of the purple Peruvian potato film (P.P film) was measured at 1.10%. After incorporating polyvinyl alcohol (PVA), forming the P.P–PVA composite film, this moisture content experienced a slight increment to 1.58%. The further integration of silver nanoparticles (AgNPs) into the P.P–PVA matrix significantly elevated the moisture content to 3.37%. This substantial rise indicates that the presence of AgNPs enhances specific interactions within the polymeric matrix, potentially increasing its hydrophilicity or contributing to a more porous structural arrangement.

The incorporation of silver nanoparticles induces distinct microstructural changes in the film matrix that enhance its practical performance. The resulting increase in equilibrium-moisture retention helps preserve a product’s intrinsic freshness, texture, and sensory quality during extended storage. Moreover, the slight rise in matrix porosity produced by AgNP addition can promote a regulated exchange of water vapor, mitigating both unwanted condensation and excessive dehydration. Such moisture-management capabilities are especially valuable in food-packaging applications, where maintaining an optimal humidity balance is essential for prolonging shelf life, safeguarding product safety, and sustaining overall consumer acceptance.

## 4. Conclusions

The incorporation of AgNPs into P.P–PVA films dramatically enhances their performance as intelligent food-packaging sensors. AgNPs act as plasmonic boosters that sharpen the pH-responsive color transition; the CIELab a* coordinate shifts from +36.1 (vivid red) to –0.1 (neutral green) while overall luminance (L*) remains virtually unchanged, ensuring clear, real-time readability. FTIR spectra confirm that the characteristic hydroxyl and carbonyl bands of both starch and PVA are preserved, demonstrating a homogeneous polymer–nanoparticle network free of chemical degradation. Moreover, AgNPs’ incorporation elevates the equilibrium moisture content from 1.10% to 3.37%, a consequence of nanoparticle-induced micro-porosity that enhances hydrophilicity and helps maintain product freshness. Taken together, these AgNP-driven enhancements endow the P.P–PVA composite with superior colorimetric sensitivity, structural stability, and barrier tunability, positioning it as a robust, multifunctional candidate for real-time food-quality monitoring.

## Figures and Tables

**Table 1:** Color parameters (L*, a*, b*) of the anthocyanin extract in buffer solutions with pH values ranging from 1.0 to 10.0.

Color Parameters				
pH Value	$L^{*}$	$a^{*}$	$b^{*}$	ΔE
pH 1.0	17.8	41.7	−5.2	86.4
pH 2.0	24.2	34.1	−5.8	77.1
pH 3.0	30.1	15.8	0.5	65.0
pH 4.0	42.7	5.8	2.4	50.9
pH 5.0	38.6	2.9	5.3	55.1
pH 6.0	3.4	13.8	2.8	90.8
pH 7.0	1.2	4.2	−12.0	92.0
pH 8.0	4.7	−8.7	−7.4	88.4
pH 9.0	46.7	−1.6	14.9	49.7
pH 10.0	30.1	−1.9	20.6	67.3

**Table 2:** Moisture content of each film and their color parameters across a range of buffer pH values.

Film	Moisture Content (%)	pH	Color Parameters				
P.P	1.10		$L^{*}$	$a^{*}$	$b^{*}$	ΔE	
		1	26.8	17.1	3.7	68.9	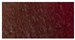
		4	28.6	12.9	2.4	65.8	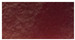
		7	30.3	10.2	11.8	65.4	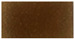
		10	29.2	−1.6	17.4	67.1	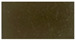
P.P–PVA	1.58						
		1	33	32.2	7.6	69.4	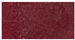
		4	37.5	19.5	8.9	60.4	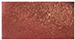
		7	40.1	13.8	14.6	57.8	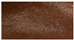
		10	31.4	0.6	7.1	62.3	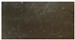
P.P–PVA–AgNP	3.37						
		1	34.3	36.1	10.8	70.9	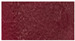
		4	37.8	24.3	16.1	63.8	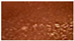
		7	37.8	12.1	16.9	60.3	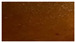
		10	33.1	−0.1	13.5	62.1	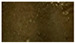

## Data Availability

The original contributions presented in this study are included in the article. Further inquiries can be directed to the corresponding authors.
